# A 12-month randomized controlled trial evaluating erythritol air-polishing versus curette/ultrasonic debridement of mandibular furcations in supportive periodontal therapy

**DOI:** 10.1186/s12903-021-01397-3

**Published:** 2021-01-21

**Authors:** Ingvild M. Ulvik, Terje Sæthre, Dagmar F. Bunæs, Stein Atle Lie, Morten Enersen, Knut N. Leknes

**Affiliations:** 1 Faculty of Medicine, Department of Clinical Dentistry, University of Bergen, Aarstadveien 19, 5009 Bergen, Norway; 2 Faculty of Dentistry, Institute for Oral Biology, University of Oslo, Oslo, Norway

**Keywords:** Air-polishing, Erythritol, Furcation involvement, Periodontal maintenance, Visual analogue scale

## Abstract

**Background:**

Due to complex morphology and limited access, the cleaning of the furcation area is extremely challenging. Therefore, novel therapeutic approaches need to be tested to potentially overcome debridement limitations. The aim of the present prospective 12-month study was to compare clinical and microbiological effects following erythritol air-polishing versus conventional mechanical debridement of furcation defects in a cohort of periodontal maintenance patients.

**Methods:**

Twenty patients with grade II mandibular molar furcation defects volunteered to enroll in this single-centre, examiner masked, randomized controlled trial. In a split-mouth study design, two furcation sites in each patient were randomly assigned to either receive subgingival debridement using erythritol air-polishing (test) or conventional ultrasonic/curette debridement (control) at baseline, and at 3, 6, 9 and 12 months. Probing depth, clinical attachment level and bleeding on probing were recorded at 3-month intervals. Subgingival microbiological samples obtained at baseline, 6 and 12 months were analyzed using checkerboard DNA–DNA hybridization. Discomfort from treatment was scored at 12 months using a visual analogue scale. The differences between treatments, and time-points, were tested using multilevel analysis (mixed effect models and robust variance estimates).

**Results:**

A significant reduction in probing depth took place following both treatments (*p* < 0.001). Control sites experienced a significant mean gain in clinical attachment level of 0.5 mm (± 0.2) (*p* = 0.004), whereas a non-significant gain of 0.4 mm (± 0.3) was observed at test sites (*p* = 0.119). At 6 months, a significant between-treatment difference of 0.8 mm (± 0.4) was observed in favor of the control (*p* = 0.032). No significant between-treatment differences were observed in microbial load or composition. Notably, at 12 months patients experienced significantly less discomfort following air-polishing compared with control (*p* = 0.001).

**Conclusions:**

The 12-month observations indicate that erythritol air-polishing and conventional mechanical debridement both support clinical improvements. A significant between-treatment difference in clinical attachment level was, however, detected in favour of control debridement at 6 months. In terms of patient comfort, erythritol air-polishing is superior.

*Trial Registration*: The clinical trial was retrospectively registered in ClinicalTrial.gov with registration NCT04493398 (07/28/2020).

##  Background

Accumulation of bacterial deposits on teeth is the primary cause of periodontitis. Non-surgical and supportive periodontal therapy (SPT) consist of mechanical debridement of microbial biofilm and dental calculus, combined with oral hygiene instructions. Disruption of microbial biofilm and removal of calculus should be performed with minimal damage to the root surface, soft tissues, and with limited patient discomfort. Traditionally, periodontal debridement is accomplished using curettes, sonic or ultrasonic scalers, all presenting comparable outcomes [1, 2]. However, periodic root instrumentation may lead to dental hard tissue [3–6] and soft tissue damage [7], and sensitivity due to exposure of dentinal tubules [8–10]. Air-polishing using low abrasive glycine or trehalose powder has been shown to reach similar clinical outcomes as hand and ultrasonic instrumentation, but with less hard tissue loss [11–16]. Moreover, air-polishing provides superior outcomes relative to patient comfort and time efficiency [7, 11, 12, 14].

Recently, a low abrasive erythritol powder with comparable physical properties to glycine air-polishing powder was introduced for subgingival air-polishing [17]. Erythritol, a non-toxic, chemically neutral and completely water-soluble polyol is widely used in food industry as an artificial sweetener. Two studies comparing conventional mechanical debridement with erythritol air-polishing, reported similar results in SPT relative to clinical and microbiological outcomes [18, 19]. Such observations are also reflected in a systematic review concluding that air-polishing systems as a monotherapy are comparable to conventional therapy in patients undergoing SPT in single- and multi-rooted teeth without furcations [20]. Moreover, inhibitory effects on pathogenic bacteria including *Porphyromonas gingivalis* have also been observed [21].

To our knowledge, no prospective studies investigating the benefit of repeated subgingival debridement with a low abrasive erythritol air-polishing system in molar furcation defects during SPT have been reported. The objective of this 12-month prospective study was to compare clinical and microbiological effects following an erythritol air-polishing system vs. conventional mechanical debridement of furcation defects in a cohort of periodontal maintenance patients.

## Methods

The study protocol and informed consent following the Helsinki Declaration of 1975 (version 2008) was approved by the Medical Research Ethics Committee (2016/793), University of Bergen, Norway. The study was conducted as a randomized controlled trial with a split-mouth study design. Participating subjects read and signed the informed consent prior to enrolling in the study. The CONSORT guidelines were followed.

### Prestudy calibration and training

Two operators performed the clinical aspects of this study. Author TS, masked to treatment assignments, performed all clinical recordings and sampling, author IU, unaware of previously recorded data, performed all treatments.

A calibration exercise was performed to obtain intra-examiner reproducibility for the primary outcome variables probing depth (PD) and clinical attachment level (CAL). In a sample of 10 patients, PD and CAL were recorded twice, 1 day apart, at six sites per tooth. Intraclass correlation coefficients (ICCs) were calculated separately for each site. ICC for repeated measures ranged between 0.87 and 1.00 for PD and between 0.88 and 1.00 for CAL. The calibration exercise also included the secondary outcome variable bleeding on probing (BoP). The Cohen’s kappa test displayed 83% agreement for the two pairs of recordings with a corresponding kappa value of 0.65.

As part of the research protocol, IU was trained in proper use of the air-polishing device and completed a pilot study in 13 patients.

### Sample size

The sample size estimation was based on change in PD. A difference of 0.5 mm was considered clinically relevant [22]. Standard deviation of the difference between repeated PD measurements from the intra-calibration exercise was 0.5 mm. A power analysis based on 20 subjects and with the level of significance (α) set to 0.05, resulted in 98.9% power to detect a true difference of 0.5 mm.

### Study subjects

**Fig. 1:**
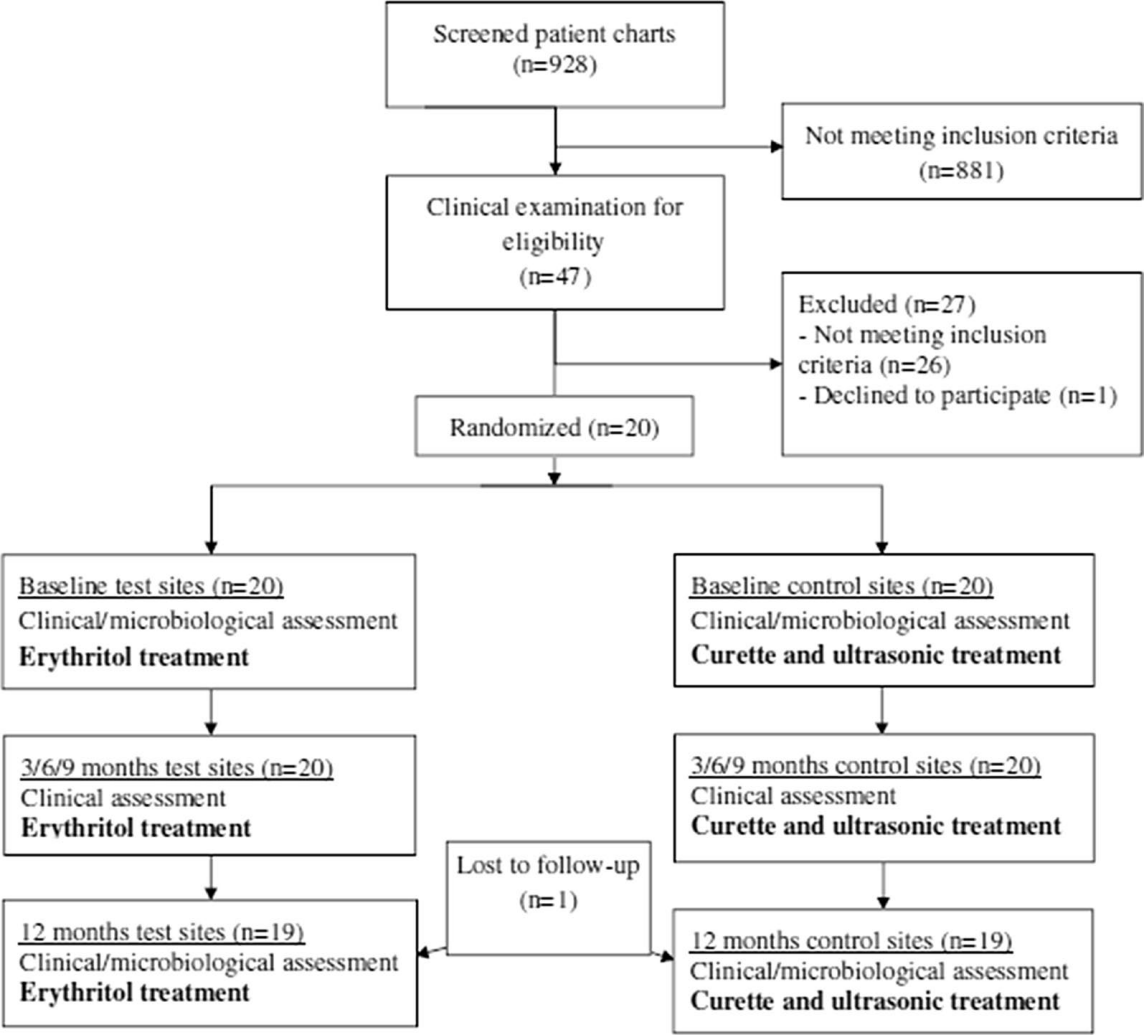
Flowchart of the study

Study subjects were recruited among patients managed in the Department of Clinical Dentistry, Section of Dental Hygiene and Section of Periodontology, University of Bergen SPT program June 2015 through June 2016. Inclusion criteria mandated 30–80-year old healthy subjects having received SPT every 3–6 months for 2–3 years following periodontal therapy, having bilateral non-mobile, fully erupted mandibular first, second or third molars with degree II furcation defects, and PD ≥ 4 mm with bleeding on probing (BoP) or pus. Following clinical examination for eligibility and medical status, 20 patients were enrolled (Fig. 1). The previous periodontal diagnosis of all included patients was moderate or severe chronic periodontitis [23].

Exclusion criteria were mobile mandibular molars, molars with clinical or radiographic evidence of supra-/subgingival calculus or apical pathology, use of systemic antibiotics within 6 months or SPT within 3 months of study, any current medical condition affecting periodontal treatment or use of the abrasive air-polishing device. Subjects with diabetes, cancer, HIV/aids, acute infections, disorders that may compromise wound healing, or pregnant were also excluded.

### Treatments

Following baseline examination, mandibular jaw quadrants were randomized (coin toss controlled by the study supervisor) to either receive debridement using the erythritol powder/air-polishing system (test) or conventional ultrasonic/curette instrumentation (control) using a split-mouth study design. Sequence of treatments was randomized in a similar fashion. Treatments were delivered at baseline, and repeated at 3, 6, 9 and 12 months. Test sites thus received root debridement using the low abrasive erythritol powder (Air-flow powder plus®, EMS, Nyon, Switzerland) applied through a Perio-Flow hand piece connected to an airflow unit (Air-Flow Master®, EMS, Nyon, Switzerland). The hand piece was fitted with a nozzle for subgingival delivery directing the power/air jet perpendicular to the root surface at the water exit at the tip of the nozzle. The nozzle was inserted to the apical aspect of furcation sites with PD ≥ 4 mm using striking movements over the furcation area for 5 s [12]. Sites adjoining the test site with PD ≥ 4 mm were similarly treated.

Control sites were debrided using an ultrasonic scaler (Piezon Master 400 Perio Slim Tip®; Electro Medical System, Nyon, Switzerland) with power set at 75% and water as coolant, and root planed with sharp curettes (Gracey SAS, Hu-Friedy, Chicago, IL, USA). Treatment of test and control sites were carried out without anesthesia.

Following treatment of test and control sites, remaining teeth were debrided with ultrasonic and hand instruments and polished using polishing paste delivered in a rotating rubber cup. For non-experimental sites, local anesthesia was used as needed. Based on the percentage of tooth surfaces with visible plaque following staining with disclosing solution, the patients received individualized oral hygiene instruction at each appointment. Patients were returned to their regular SPT upon completion of study in which site-specific adjunctive therapy was continuously considered.

### Clinical assessment

Before clinical examination, radiographs of the test and control teeth were used to assess vertical bone loss and to rule out apical pathology and supra- or subgingival calculus. The following clinical parameters were recorded at baseline, and at 3, 6, 9 and 12 months of study: PD as the distance from the gingival margin to the probable base of the pocket in mm; CAL as the distance in mm from the cemento-enamel junction or the margin of a dental restoration to the probable base of the pocket. Local probing at test and control sites and full mouth PD and CAL at six sites per tooth were recorded to the closest mm using a periodontal probe (PCP, UNC 15, Hu-Friedy, Chicago, IL, USA). BoP was recorded as present upon gentle probing to the base of the pocket [24]. Full mouth gingival bleeding was recorded as percentage of sites showing BoP assessed at four sites per tooth, including local bleeding at test and control sites. Full mouth dental plaque scores were recorded as the percentage of tooth surfaces with visible plaque following staining with disclosing solution assessed at four sites per tooth [25]. Presence or absence of plaque at control and test sites were also recorded [25]. Furcations were classified at baseline and at follow-up examinations using a curved scaled Nabers furcation probe marked at 3 mm intervals (PQ2N; HU-Friedy) according to horizontal classification criteria [26]. Furcation defects, featuring horizontal loss of periodontal support > 3 mm into the furcation but not encompassing the total width of the furcation area, were classified as degree II [26]. Vertical attachment loss at furcation site was assessed using a periapical radiograph and clinical probing depths/CALs [27].

### Gingival crevicular fluid assessments

Gingival crevicular fluid (GCF) was recorded at baseline, and at 6 and 12 months [28]. Briefly, furcation sites were isolated with cottons rolls, cleaned for supragingival plaque, and air-dried. A perio paper strip was then placed 1–2 mm into the orifice of the site and left in place for 30 s. Next, the perio strip was inserted into the Periotron 8000® (Oraflow, Smithtown, NY, USA) calibrated to estimate the volume of GCF collected.

### Microbiological assessments

At baseline, and at 6 and 12 months the supragingival area above the furcation site was wiped clean using sterile cotton pellets. Three sterile paper points were then inserted into the pocket of the furcation site. The paper points were kept in place 20 s [29] removed and immersed into pre-reduced, anaerobic transport medium (PRAS; Dental Transport Medium, Morgan Hill, CA, USA). Sample tubes, separately pooled by treatment, were sent to Microbiological Diagnostic Service, Institute of Oral Biology, University Oslo, Norway for analysis using checkerboard DNA-DNA hybridization [30, 31]. Bacterial samples were analyzed for qualitative and quantitative detection of”red complex” species *P. gingivalis*, *Treponema denticola* and *Tannerella forsythia* [32] as well as *Aggregatibacter actinomycetemcomitans*, *Prevotella intermedia*, *Fusobacterium nucleatum subsp. polymorphum*, *Fusobacterium nucleatum subsp. vincentii*, *Fusobacterium nucleatum subsp. nucleatum, Parvimonas micra* and *Prevotella nigrescens*.

### Pain experience assessments

Visual analogue scale (VAS) scores were used to estimate patient discomfort experienced during test and control treatment [33]. Scoring was performed at 12 months following completion of the debridement with 0 = “no pain” and 100 = “worst pain I can imagine”.

### Statistical analysis

Data were entered into MS-Excel (Microsoft, Redmond, WA, USA) proofed for errors and then imported into Stata, version 15 (StataCorp, College Station, TX, USA). All analyses were performed by a statistician (SAL) who had not taken part in data collection or treatments. Primary clinical outcome variables were changes in PD and CAL. BoP, GCF, total number bacteria and VAS scores were defined as secondary outcome variables.

Summary statistics (means ± SEM) for the clinical variables were calculated for the test and control at baseline, and at 6 and 12 months. Due to the repeated nature of data, multilevel analysis (mixed effect models) taking into consideration incomplete data at 12 months was applied to analyze the data at patient and tooth level. Time and treatment were considered fixed factors. Mixed models were applied for both primary and secondary outcome variables.

For testing differences in microbial composition at test and control sites harboring different proportions of bacteria > 10^5^ at baseline, and at 6 and 12 months, logistic regression models with robust standard error were applied. VAS scores were analyzed using ordinary linear regression models with robust standard error. The level of significance was set at 0.05.

## Results

A total of 928 patient charts were screened, 881 charts excluded not meeting study inclusion criteria (Fig. 1). The remaining 47 patients were clinically examined and 20 patients meeting study inclusion criteria were enrolled (Table 1). The study group included 14 males and six females, mean age 61 years, range 39–78 years. Nine patients reporting daily smoking during the last 5 years were classified as smokers. Thirteen buccal and seven lingual furcation sites were debrided using erythritol powder/air-polishing system (test), 16 buccal and 4 lingual were debrided using conventional ultrasonic/curette techniques (control). This between-difference in location of included furcation sites was non-significant (*p* = 0.479). Nineteen patients completed the 12-month study. One patient had moved from the area by the 9-month examination.

**Table 1:** Patient and site characteristics

Patient	Gender	Age	Smoker	Test site	Control site
1	Male	66	Yes	36 buccal	46 buccal
2	Female	71	Yes	47 buccal	36 buccal
3	Male	45	Yes	48 lingual	37 lingual
4	Male	78	No	46 buccal	36 buccal
5	Female	54	No	46 lingual	37 buccal
6	Female	69	No	46 buccal	36 buccal
7	Male	60	No	36 lingual	47 buccal
8	Male	39	Yes	48 buccal	37 buccal
9	Female	53	No	37 lingual	47 buccal
10	Male	77	No	36 buccal	46 buccal
11	Male	72	No	47 buccal	37 buccal
12	Male	48	Yes	36 lingual	46 lingual
13	Male	67	No	47 lingual	36 buccal
14	Male	54	No	37 buccal	47 buccal
15	Female	60	Yes	36 lingual	46 buccal
16	Male	45	Yes	47 buccal	37 lingual
17	Male	78	No	47 buccal	36 lingual
18	Female	54	Yes	36 buccal	46 buccal
19	Male	70	No	46 buccal	37 buccal
20	Male	58	Yes	37 buccal	47 buccal

### Clinical observations

Patient level clinical observations are summarized in Table 2. Significant reductions were observed for PD, CAL, BoP (all *p* < 0.001) and for Plaque (*p* = 0.010) from baseline to 6 months (*p* < 0.001) and from baseline to 12 months (*p* = 0.009). The proportion of PD ≤ 4 mm (pocket closure) increased from 87.7% at baseline to 93.1% and 95.1% at 6 and 12 months, respectively. For moderate (4–6 mm) and deep pockets (> 6 mm) the corresponding decreased from baseline to 6 and 12 months were 10.4%, 6.1, 4.3 and 1.9, 0.8, 0.6, respectively. Between 6 and 12 months only CAL (*p* < 0.001) and BoP (*p* < 0.002) were significantly reduced.

**Table 2:** Patient level mean (± SEM) recordings for the clinical parameters at baseline, 6, 9, and 12 months and mean change (Δ) from baseline and 6 months

	Baseline	3 months	6 months	9 months	12 months	Δ_0–6_	*p*	Δ_6–12_	*P*	Δ_0–12_	*p*
PD	3.2 ± 0.1	2.9 ± 0.1	2.8 ± 0.1	2.7 ± 0.1	2.7 ± 0.1	− 0.4 ± 0.0	< 0.001	− 0.2 ± 0.0	0.094	− 0.5 ± 0.1	< 0.001
CAL	4.0 ± 0.2	3.7 ± 0.2	3.6 ± 0.2	3.5 ± 0.2	3.5 ± 0.2	− 0.3 ± 0.0	< 0.001	− 0.2 ± 0.0	< 0.001	− 0.5 ± 0.1	< 0.001
Plaque	56.1 ± 4.3	45.5 ± 4.3	46.5 ± 4.5	45.6 ± 4.8	44.6 ± 5.2	− 9.6 ± 3.8	0.010	− 1.8 ± 3.8	0.629	− 11.5 ± 4.4	0.009
BoP	54.2 ± 4.1	38.4 ± 4.1	35.6 ± 4.1	32.3 ± 4.1	25.3 ± 4.1	− 18.7 ± 3.3	< 0.001	− 10.3 ± 3.3	0.002	− 28.9 ± 3.3	< 0.001

Probing depth (PD) and clinical attachment level (CAL) in mm; plaque and bleeding on probing (BoP) in %

**Fig. 2:**
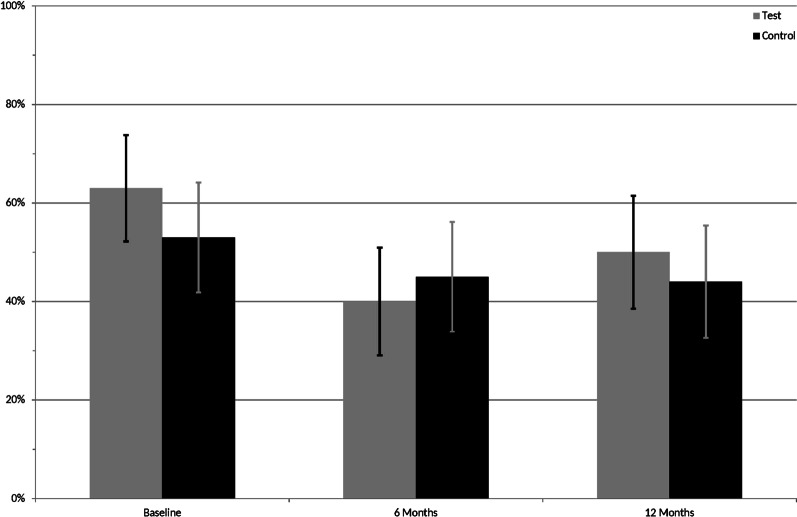
Percent test and control furcation sites showing plaque at baseline, 6 and 12 months

**Fig. 3:**
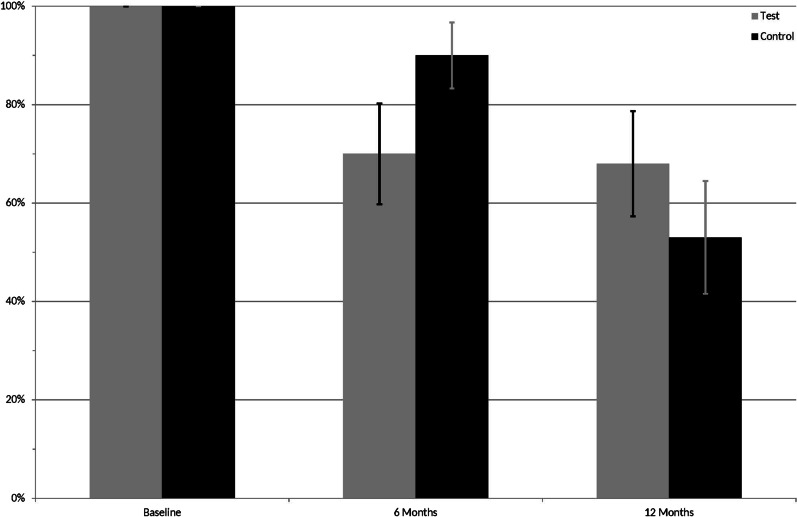
Percent test and control furcation sites showing bleeding on probing at baseline, 6 and 12 months

At furcation site level, the clinical status of test and control in terms of PD (*p* = 0.468), CAL (*p* = 0.221), GCF (*p* = 0.937), Plaque (*p* = 0.634) and BoP (*p* = 1.000) were homogeneous and revealed no significant difference at baseline (Table 3, Figs. 2, 3). A statistically significant reduction in PD was observed in both test and control from baseline to 6 months (both *p* < 0.001) and baseline to 12 months (both *p* < 0.001; Table 3). For test sites, the proportion of PD ≤ 4 mm (pocket closure) increased from 60.0% at baseline to 65.0% and 79.0% at 6 and 12 months, respectively. A corresponding increase was observed for control sites from 60.0% at baseline to 80.0% and 84.2% at 6 and 12 months. The difference in mean PD between 6 and 12 months, was not significant (*p* = 0.331 and *p* = 0.534 for test vs. control). No significant between-treatment differences were observed for any timepoint.

**Table 3:** Site furcation level mean (± SEM) recordings for the clinical parameters and gingival crevicular fluid volume at baseline, and at 6 and 12 months and mean change (Δ) from baseline and 6 months

	Baseline	*p*	6 months	*p*	12 months	*p*	Δ_0–6_	*p*	Δ_6–12_	*p*	Δ_0–12_	*p*
PD												
Test	4.8 ± 0.4		4.0 ± 0.4		3.7 ± 0.4		− 0.9 ± 0.2	< 0.001	− 0.2 ± 0.2	0.331	− 1.0 ± 0.2	< 0.001
Control	4.6 ± 0.2		3.5 ± 0.3		3.4 ± 0.3		− 1.05 ± 0.2	< 0.001	− 0.1 ± 0.2	0.534	− 1.2 ± 0.3	< 0.001
Difference	0.2 ± 0.3	0.468	0.5 ± 0.4	0.192	0.4 ± 0.4	0.232	0.20 ± 0.3	0.454	− 0.0 ± 0.3	0.901	0.2 ± 0.3	0.540
CAL												
Test	5.5 ± 0.4		5.2 ± 0.4		5.1 ± 0.4		− 0.3 ± 0.2	0.175	− 0.1 ± 0.2	0.502	− 0.4 ± 0.3	0.119
Control	5.0 ± 0.2		4.4 ± 0.3		4.5 ± 0.3		− 0.6 ± 0.1	< 0.001	0.1 ± 0.1	0.584	− 0.5 ± 0.2	0.004
Difference	0.5 ± 0.4	0.221	0.8 ± 0.4	0.032	0.6 ± 0.4	0.097	0.3 ± 0.2	0.107	− 0.2 ± 0.2	0.404	0.2 ± 0.2	0.448
GCF												
Test	79.5 ± 7.5		57.3 ± 7.5		44.0 ± 7.7		− 22.2 ± 11.0	0.044	− 14.6 ± 11.2	0.171	− 36.7 ± 11.2	0.001
Control	78.8 ± 7.5		55.1 ± 7.5		45.0 ± 7.7		− 23.7 ± 7.6	0.002	− 10.6 ± 7.7	0.171	− 34.2 ± 7.7	< 0.001
Difference	0.7 ± 8.8	0.937	2.2 ± 8.8	0.804	− 1.0 ± 9.1	0.917	− 1.5 ± 12.5	0.905	3.2 ± 12.7	0.804	1.7 ± 12.7	0.897

Probing depth (PD) and clinical attachment level (CAL) in mm; gingival crevicular fluid (GCF) in µL

Control furcation sites showed a significant gain in CAL from baseline to 6 months (*p* < 0.001) and from baseline to 12 months (*p* = 0.004), corresponding gains for the test furcation sites did not reach statistical significance (*p* = 0.175 and *p* = 0.119; Table 3). The difference in CAL between 6 and 12 months was not significant for either treatment (*p* = 0.502 and *p* = 0.584 for test vs. control). At 6 months there was a significant between-treatment difference of 0.8 mm (± 0.4) in favor of the control (*p* = 0.032), a tendency also observed at 12 months (0.6 mm ± 0.4; *p* = 0.097). For each treatment approach no change in horizontal furcation involvement was observed during the observation period. At 12 months, all furcations showed horizontal attachment loss > 3 mm and were classified as degree II (not tabulated).

Mean GCF volume was reduced from 79.5 to 57.3 µL in test furcation sites (*p* = 0.044) and from 78.8 to 55.1 µL in control sites (*p* = 0.002) from baseline to 6 months. The mean GCF volume was further reduced to 44.0 µL in test sites and 45.0 µL in control sites at 12 months. For both treatments the reduction from baseline to 12 months was statistically significant (*p* < 0.001). No significant between-treatment differences were observed for any timepoint (Table 3).

At baseline, 63% of the test furcation sites and 53% of the control furcation sites showed visible plaque following staining. By 6 months, the corresponding observations were 40% and 45%, and at 12 months 50% and 44% (Fig. 2).

Number of furcation sites exhibiting BoP are shown in Fig. 3. At baseline, all sites (100%, inclusion criteria) showed BoP, whereas at 6 months the number decreased to 70% and 90%, and at 12 months to 68% and 53% for test and control sites, respectively.

### Microbiological observations

**Fig. 4:**
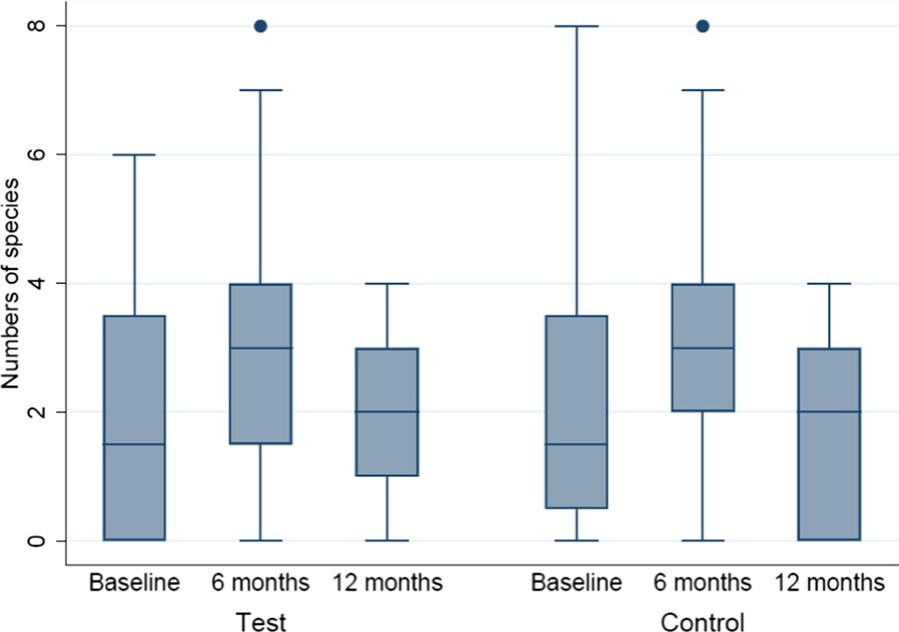
Numbers of bacterial species in test and control furcation sites

Total mean numbers of species present in test and control furcation sites at baseline were 1.95 and 2.25, respectively. Corresponding numbers at 6 and 12 months were 3.10, 3.15 and 2.21, 1.76 (Fig. 4). For test sites, the observed within-treatment increase from baseline to 6 months was significant (*p* = 0.025), whereas the decrease from 6 to 12 months was non-significant (*p* = 0.079). The corresponding within-treatment increase for control sites from baseline to 6 months was non-significant (*p* = 0.083), whereas the decrease from 6 to 12 months was significant (*p* = 0.009). No significant between-treatment differences were detected at baseline (*p* = 0.523) or at 6 (*p* = 0.706) and 12 (*p* = 0.334) months.

**Fig. 5:**
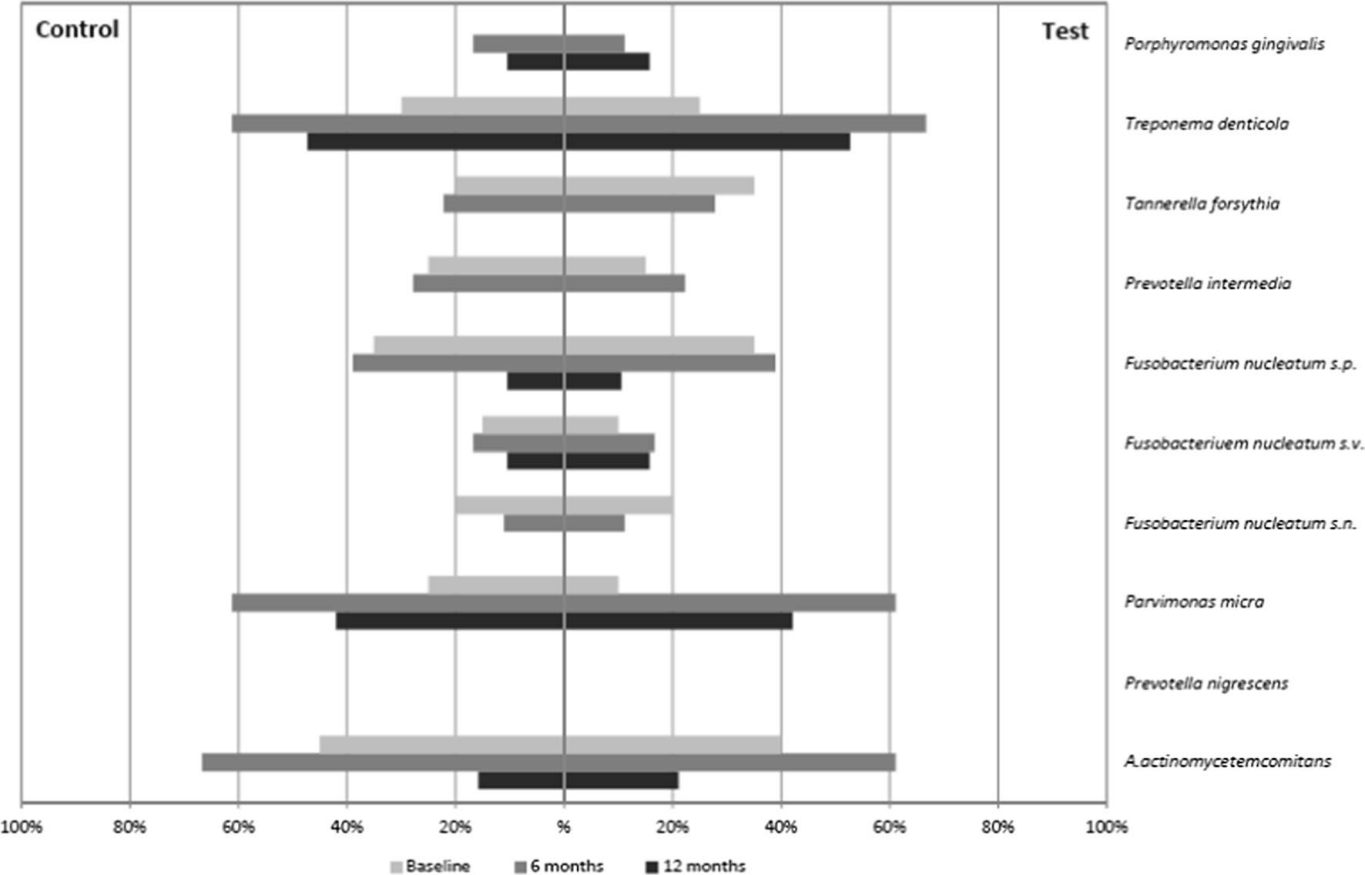
Percent test and control furcation sites harboring targeted bacterial species > 10^5^ at baseline, and at 6 and 12 months

The percentages of test and control furcation sites positive for analyzed bacterial species > 10^5^ at baseline, at 6 and 12 months are presented in Fig. 5. *P. micra* and *T. denticola* displayed a significant increase from baseline to 6 months for both treatments (*p* = 0.013 vs. *p* = 0.023). For all other species, no significant between- or within-treatment differences were detected at any observation interval.

### Treatment discomfort

Pain experience during treatment scored at 12 months using VAS showed significantly lower mean scores (less pain) for the erythritol air-polishing system (13.3) compared with conventional mechanical debridement (13.3 vs. 32.6; *p* = 0.001). For test as well as for control treatment, no significant overall interaction was observed between site-specific clinical parameters and VAS-scores at patient level (*p* > 0.05). There was a non-significant increase in VAS-scores with increasing CAL for both treatments (test: *p* = 0.61, control: *p* = 0.12, not tabulated). No adverse events were observed or reported for any of the treatment protocols.

## Discussion

The objective of this 12-month prospective study was to compare clinical and microbiological effects following an erythritol air-polishing system versus conventional mechanical debridement of furcation defects in a cohort of periodontal maintenance patients. The observations herein suggest that conventional mechanical debridement and erythritol air-polishing both support clinical improvements. A significant between-treatment difference in CAL was observed at 6 months in favor of conventional debridement. Notably, at 12 months the erythritol air-polishing system was deemed the most comfortable intervention by the patients.

Enrolled subjects had regularly participated in a SPT program prior to the study. The overall compliance with oral hygiene instruction was suboptimal as demonstrated by the consistently elevated plaque and bleeding scores. Another contributing factor might be diet consumption characterized by high content of processed high-glycemic carbohydrates and low content of micronutrients and fibres, both promoting plaque accumulation and gingival inflammation [34–36]. Another 12-month randomized, controlled trial evaluated the effect of Er:YAG laser treatment of periodontal sites with recurring inflammation in maintenance patients recruited from a university environment [37]. In that study, the numbers of experimental teeth exhibiting supragingival plaque and BoP were high (> 77%) throughout the study with no significant difference between test and control sites. Similar high plaque and bleeding scores of more than 55% were consistently reported in a 6-month randomized clinical trial evaluating the effect of professional tooth cleaning prior to non-surgical periodontal therapy in private practice [38]. Lack of compliance may reflect weariness and loss of motivation after years of comprehensive periodontal therapy and repeated mechanical debridement during maintenance care [37]. Conceptually, it is more challenging to obtain clinical improvement in residual lesions than in previously untreated pockets. Interestingly, dental biofilm may account for only 20% of the direct risk for developing periodontitis [39, 40]. This may indicate that the outcome of subgingival debridement is not only influenced by the efficacy of instrumentation and supragingival biofilm control, but by systemic patient factors as well [38].

In a split-mouth study design, each patient serves as his/her own control. This approach nullifies the impact of inter-individual variations related to patient characteristics as age, gender, systemic condition, genetic susceptibility, smoking status, oral hygiene and by such obtain a more powerful estimation of treatment effects. Subanalyses for the primary outcome variables PD and CAL revealed that the inclusion of smokers did not interfere with the site-specific treatment outcomes. With a parallel group study design, biological variations among subjects in disease and treatment response might be greater than differences between treatments. However, there are also some drawbacks inherent in split-mouth study design. First, it may lead to biased treatment efficacy due to carry across effects. Second, the recruitment of patients is challenging, because of the required symmetrical disease distribution [41–43].

Furcation involved molars have been shown to respond less favorable to scaling and root planing, more than likely due to difficulty to effectively disrupt the subgingival biofilm [44]. That in 81% of molars the furcation entrance measures 1 mm or less restricting the access to conventional mechanical debridement signifies this dilemma [45]. Further, anatomical peculiarities including mesial/distal root concavities should be expected and narrow lingual furcation entrances [46] adding complexity to effective furcation debridement using any protocol. In the present study, buccal and lingual furcations were almost equally distributed between the test and control and an unbalanced treatment effect due to anatomical variations was probably minimized.

Both test and control sites showed significant reduction in PD from baseline to 12 months without significant differences between protocols. This observation is consistent with previous reports comparing low abrasive air-polishing with conventional mechanical debridement [13–15, 17–19]. For example, in a split-mouth study, 50 patients were monitored at 3-month intervals over 12 months. Sites presenting with PD > 4 mm were randomly allocated either to subgingival air-polishing with erythritol or ultrasonic debridement. For both treatments, the numbers of periodontal pockets at 12 months were significantly reduced compared with baseline [19].

Control sites showed statistically significant CAL improvements from baseline to 6 months and from baseline to 12 months. Corresponding improvements in test sites, however, did not reach statistical significance. In a randomized clinical trial assessing the efficacy and safety of glycine air-polishing in moderate/deep periodontal pockets [11–13, 47] and of erythritol powder in residual pockets [19], the 5-s treatment time/site adopted in the present study was advocated. The subgingival nozzle tip used in this study is not specially designed to immediately access subgingival furcation’s complex horizontal/vertical anatomy and inherent concavities [12]. Perhaps a longer treatment interval is required in furcation sites to exhaust the efficacy of the air-polishing system. Also design figurations of the subgingival nozzle tip reach into furcation defects could be considered to advance performance. Further, the fact that included patients were previously treated by dental students will increase the possibility of leaving residual subgingival calculus in the furcation and thus, favouring the outcome of a conventional treatment approach.

GCF flow from a periodontal site is influenced by the degree of inflammation in the soft tissues and extent of ulceration of the sulcular/pocket epithelium [48]. In this study, both test and control sites showed a decrease in GCF flow from baseline to 12 months consistent with other clinical follow-up studies [49, 50]. That no significant between-treatment differences were observed parallel improved PD and CAL surrogate estimates of gingival inflammation.

Microbiological observations could not reveal significant differences between test and control at any observation interval. Notably, *T. denticola* and *P. micra* of the “red” and “orange complex” [32] were significantly increased from baseline to 6 months for both protocols. Some studies comparing air-polishing with scaling and root planing reported air-polishing to be superior when microbiological sampling of the test sites was performed immediately or shortly following instrumentation [11, 13, 47]. Still others have reported results similar to those herein [12, 14]. For example, Hagi et al. [18] compared microbiological findings at baseline and 6 months following subgingival erythritol air-polishing and scaling and root planing. At 6 months, no microbiological between-treatment differences were observed. In the past, it has been demonstrated that bacterial biofilms can recolonize rapidly following subgingival instrumentation [51] and that microbiota may reach pretreatment levels within months [52]. The microbiological outcomes of the current study, derived from samples taken 3 months following treatment, may basically mirror the recolonization process.

The perception of pain during/following root instrumentation is a critical factor for optimal compliance with an SPT program. Significant lower VAS scores were observed for erythritol air-polishing compared with conventional treatment. These findings echo previous studies comparing air-polishing with conventional mechanical debridement [7, 11, 12, 14]. Patients are concerned about instrumentation time and pain. Since both the test and control herein improved the furcation health erythritol air-polishing might be an alternative to conventional debridement particularly when considering faster biofilm removal and less loss of tooth substance [53].

We acknowledge some additional limitations of the study. The sample size was relatively small and as such, results must be interpreted in perspective. Due to selection bias only including mandibular molars, the study observations should not be generalized. Further, intra-individual comparisons have their limitations as effects of local therapy may carry across to influence the outcomes in other areas of the dentition [54]. An adequate randomization method intends to minimize the likelihood that real differences in treatment outcomes are merely reflected by chance. Mandibular jaw quadrants were randomized by coin toss to either test or control debridement. However, coin toss is not recommended by the CONSORT guidelines [55] except for large samples. With the present small number of participants, possible confounding bias from unknown factors can therefore not be completely excluded. Due to limited access, the cleaning of a furcation sites on 48/38 is more challenging than on first molars. Two third molars were included in the analysis, and both were randomized to the test group. It therefore cannot be precluded that this anatomical confounder might have affected the outcomes. Regrettably, VAS scores were only recorded at 12 months. Thus, no analysis of pain experience during treatment throughout the study period can be presented.

## Conclusions

The observations suggest that conventional mechanical debridement and erythritol air-polishing both support clinical improvements. A significant between-treatment difference in clinical attachment level was, however, detected in favour of conventional debridement at 6 months. Treatments displayed similar effects on the subgingival microflora. The erythritol air-polishing system was deemed the most comfortable intervention by the patients.

## Data Availability

The datasets used and analysed during the current study and coding are available from the corresponding author on reasonable request.
